# Osmium-Based Pyrimidine Contrast Tags for Enhanced Nanopore-Based DNA Base Discrimination

**DOI:** 10.1371/journal.pone.0142155

**Published:** 2015-12-11

**Authors:** Robert Y. Henley, Ana G. Vazquez-Pagan, Michael Johnson, Anastassia Kanavarioti, Meni Wanunu

**Affiliations:** 1 Department of Physics, Northeastern University, Boston, Massachusetts, United States of America; 2 Department of Biology, Northeastern University, Boston, Massachusetts, United States of America; 3 Department of Chemistry and Chemical Biology, Northeastern University, Boston, Massachusetts, United States of America; 4 Yenos Analytical LLC, El Dorado Hills, California, United States of America; Wake Forest University School of Medicine, UNITED STATES

## Abstract

Nanopores are a promising platform in next generation DNA sequencing. In this platform, an individual DNA strand is threaded into nanopore using an electric field, and enzyme-based ratcheting is used to move the strand through the detector. During this process the residual ion current through the pore is measured, which exhibits unique levels for different base combinations inside the pore. While this approach has shown great promise, accuracy is not optimal because the four bases are chemically comparable to one another, leading to small differences in current obstruction. Nucleobase-specific chemical tagging can be a viable approach to enhancing the contrast between different bases in the sequence. Herein we show that covalent modification of one or both of the pyrimidine bases by an osmium bipyridine complex leads to measureable differences in the blockade amplitudes of DNA molecules. We qualitatively determine the degree of osmylation of a DNA strand by passing it through a solid-state nanopore, and are thus able to gauge T and C base content. In addition, we show that osmium bipyridine reacts with dsDNA, leading to substantially different current blockade levels than exhibited for bare dsDNA. This work serves as a proof of principle for nanopore sequencing and mapping via base-specific DNA osmylation.

## Introduction

The development of new DNA sequencing methods has been spurred forward by the desire for high accuracy, long read length, high throughput, and low cost. Deamer and Church originally postulated a method of DNA sequencing using nanopores [1, 2], wherein nucleotide bases are read out as a strand of DNA is driven through a nanopore. If a voltage bias is applied across a nanopore that separates two chambers of a conductive electrolyte solution, a steady-state ion current baseline is generated across the pore. When DNA is added to the grounded chamber (*cis*) individual molecules are electrophoretically threaded and driven through the nanopore, and during their passage transient ion current reductions are observed. Given the mild difference in size between the four nucleobases, each nucleotide should in theory produce a characteristic current level as it traverses an ultrathin nanopore (<1 nm thickness), from which the original DNA sequence can be read.

However, in practice this approach has been complicated by the need to slow the transport of DNA through the nanopore to speeds detectable with current patch-clamp amplifiers, as well as the need for ultrathin nanopores. A compromise from the ideal situation affords a DNA sequencing system that has been recently realized. In this system, DNA is enzymatically ratcheted through a biological nanopore embedded in an organic membrane, in a base-by-base fashion [3, 4]. However, given the finite thickness of the biological pores used, the current signal that is recorded contains information from multiple nucleotides. For example, DNA has been sequenced using Mycobacterium smegmatis porin A (MspA), which has a constriction thickness of ~0.6 nm [5]. Previous MspA experiments have shown that the current levels correspond to about four nucleotides that are in and around the constriction [3], which results in 4^4^ = 256 possible current level combinations. The large number of current levels that must be resolved can reduce the overall accuracy of the sequencing method, especially for situations in which two sequences produce nearly degenerate current levels.

**Fig 1:**
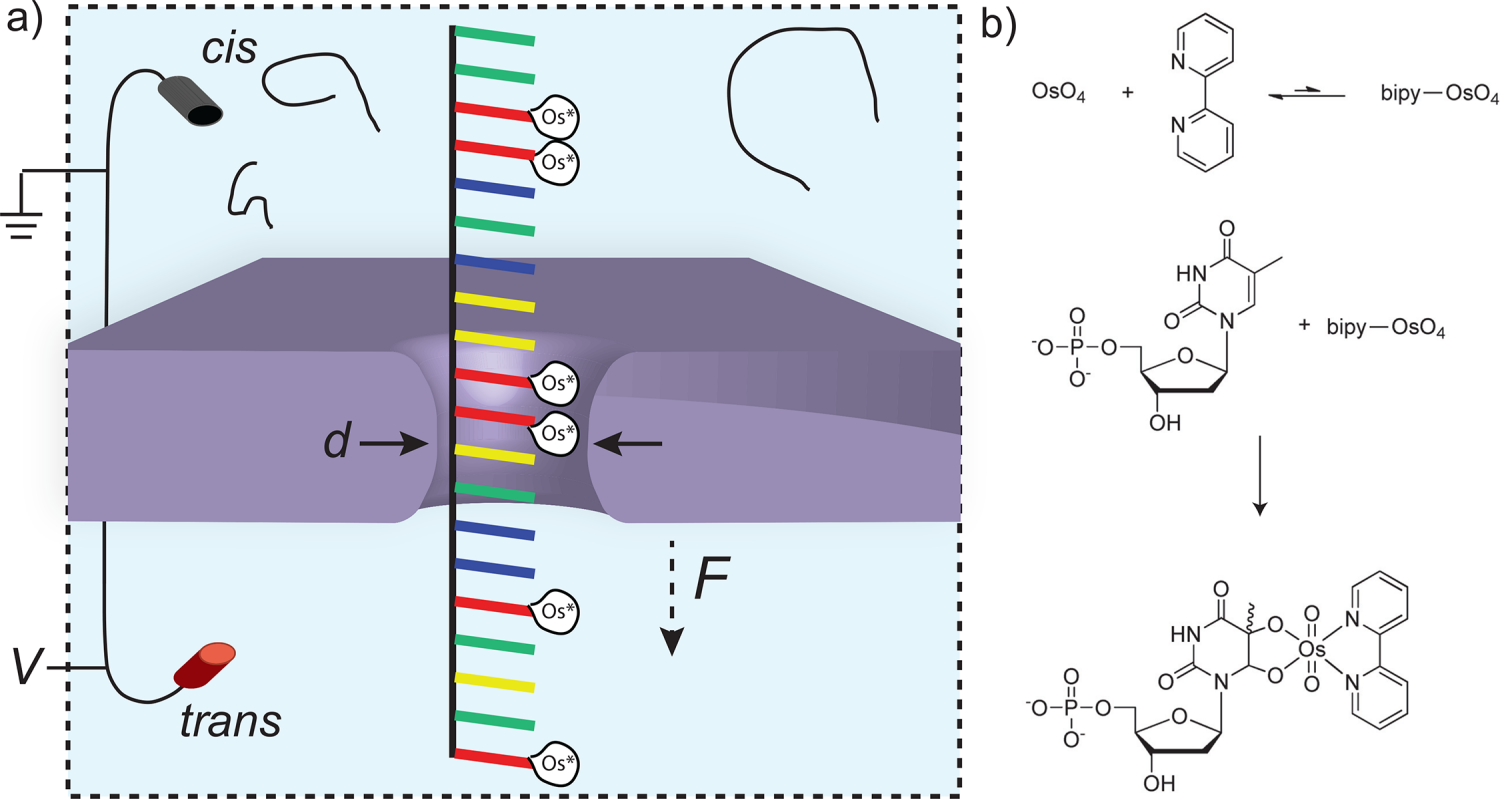
Solid-state nanopore detection of osmylated bases. **(a)** Schematic view of the nanopore ssDNA detection system. Voltage is applied across a SiN membrane containing a ~1.6 nm diameter nanopore using a pair of electrodes, leading to a steady-state ion current across the pore. When negatively charged DNA molecules are added to the *cis* chamber they are electrophoretically drawn through the nanopore to the positively charged electrode in the trans chamber. “Os*” tags are shown to represent osbipy molecules as they preferentially bind to thymidine bases (represented in red). **(b)** Reaction of OsO_4_ with bipyridine to form the bipy-OsO_4_ reactive complex. The complex then reacts with dTMP to form the osmylated adduct.

It has been suggested that the problem of chemically comparable bases could be circumvented by the addition of nucleotide-specific chemical modifications [6, 7], as in Maxam-Gilbert sequencing [8]. One such candidate modification is the reaction of osmium tetroxide 2,2′-bipyridine (Osbipy) with pyrimidine bases, wherein *syn* addition of two oxygens on the osmium center occurs on the C_5_-C_6_ double bond of pyrimidine bases [9]. Osbipy selectively reacts with T, although both pyrimidines (T + C) can also be reacted under prolonged reaction conditions. Purines (A + G) remain intact under all tested conditions. Since osbipy increases the mass of the reacted base by ~400%, a large difference between reacted and unreacted base should be observed in a nanopore measurement. The osmylation-assisted nanopore sequencing method previously described proposes a stepwise method for gaining sequence information by locating all instances of each of the four nucleobases one by one [7]. First, all T bases on the template strand are osmylated and fed through the nanopore, each base is detected as being either osmylated (T) or not osmylated (other). Next, the template strand is further osmylated to label all C bases and again the template is fed through the nanopore, any position that is now osmylated where it was not previously corresponds to a C base. The process is then repeated for the complementary strand, where the C and T bases correspond G and A bases respectively on the template strand. This allows for any sequencing strategy to simply distinguish between osmylated and unosmylated bases. In the example of the MspA pore, this would reduce the possible number of signals from 4^4^ = 256 to 2^4^ = 16, which would simplify the problem by reducing the rate of errors. Here we provide a proof of principle for this concept by translocating ssDNA with varying numbers of osmylated bases through nanopores in ultrathin silicon-nitride (SiN) membranes. We show that the fractional ion current blocked by the translocating molecule (*ΔΙ/Ι*_*o*_) increases with the fraction of osmylated bases, suggesting that osmylation may be a viable contrast agent for DNA sequencing using nanopores. **Fig 1** presents a scheme of our nanopore measurements of osmylated single-stranded DNA (ssDNA), as well as the chemical details of thymidine osmylation. We note that the issue of controlled base-by-base motion of DNA through a nanopore, a critical prerequisite for sequencing, is not addressed here. Instead, we focus on on the possible merits of increasing the contrast between DNA nucleotides by chemically modifying them in a base-specific manner.

While we focus the majority of our present study on ssDNA, there are advantages to being able to detect osmylated double-stranded DNA (dsDNA). In particular, the use of osbipy as a DNA contrast agent may also have applications in DNA mapping technologies. Next-generation sequencing methods that read single molecules have proven to be extremely useful for long read length sequencing, with average read lengths of several kilobases that extend to >100 kb [10, 11]. Despite these long read lengths, a reference genome is still required in order to assemble a full eukaryotic genome from individual nanopore reads. In the absence of a reference genome, *de novo* sequencing requires complementary DNA mapping tools for accurate sequence alignment [12]. A recent report demonstrated the combination of single-molecule sequencing and single-molecule genome mapping techniques for the assembly of a human genome [13]. By enabling a high-quality assembly, these techniques can mitigate bias that arises from an overreliance on a single reference genome. Thus, a fully *de novo* approach allows for a more complete characterization of genome variation, giving us a greater understanding of the consequences of such slight variations. A nanopore-based DNA mapping technology is proposed here which is reminiscent of a DNA footprinting assay. In this method, double-stranded DNA first complexes with a sequence specific binding protein (transcription factor, zinc-finger protein, transcription activator-like effector (TALE), repeat specific protein, etc.), and the DNA/protein complex is then stained with a contrast agent that will bind to regions of the DNA that are not protein bound (e.g., the pyrimidine-reactive osbipy complex), followed by protein denaturation and its dissociation from the DNA. Readout can then be achieved by translocating the DNA through nanopores slightly wider than the DNA cross-section (~3 nm), which results in highly regular transport as the narrow pore geometry minimizes DNA self-interaction near the pore [14]. The main potential advantage of this approach for mapping is that smaller pores can be used without the need for the DNA bound proteins to remain on the DNA during the experiment. With this motivation in mind, we show here that osmylated dsDNA indeed produces a distinct signature from unlabeled dsDNA.

## Results and Discussion

To first establish the sensitivity of SiN nanopores to osmylated DNA, we fabricated nanopores in SiN substrates as previously described [15]. We then inserted chips containing nanopores into custom-made PTFE cells and sealed them using elastomeric gaskets. These chips separate two chambers filled with a buffered solution containing 0.40 M KCl, 4.8 M urea, 1 mM EDTA, and 10 mM Tris, at pH 8.0 (the presence of urea was found to decrease second structures and clogging of the nanopore). Upon establishing a stable baseline current (*I*_*o*_), we added a solution of ssDNA to the cis chamber of the cell to a final concentration of ~1 nM. We then applied a positive bias voltage across the pore in order to electrophoretically drive ssDNA molecules across the pore, where they were detected as transient reductions in the ion current through the pore (*ΔI*). Each translocation event was then analyzed to extract the fraction of the total current blocked (*ΔI/I*_*o*_) and the time that it takes the molecule to translocate the pore (*t*_*d*_).

**Fig 2:**
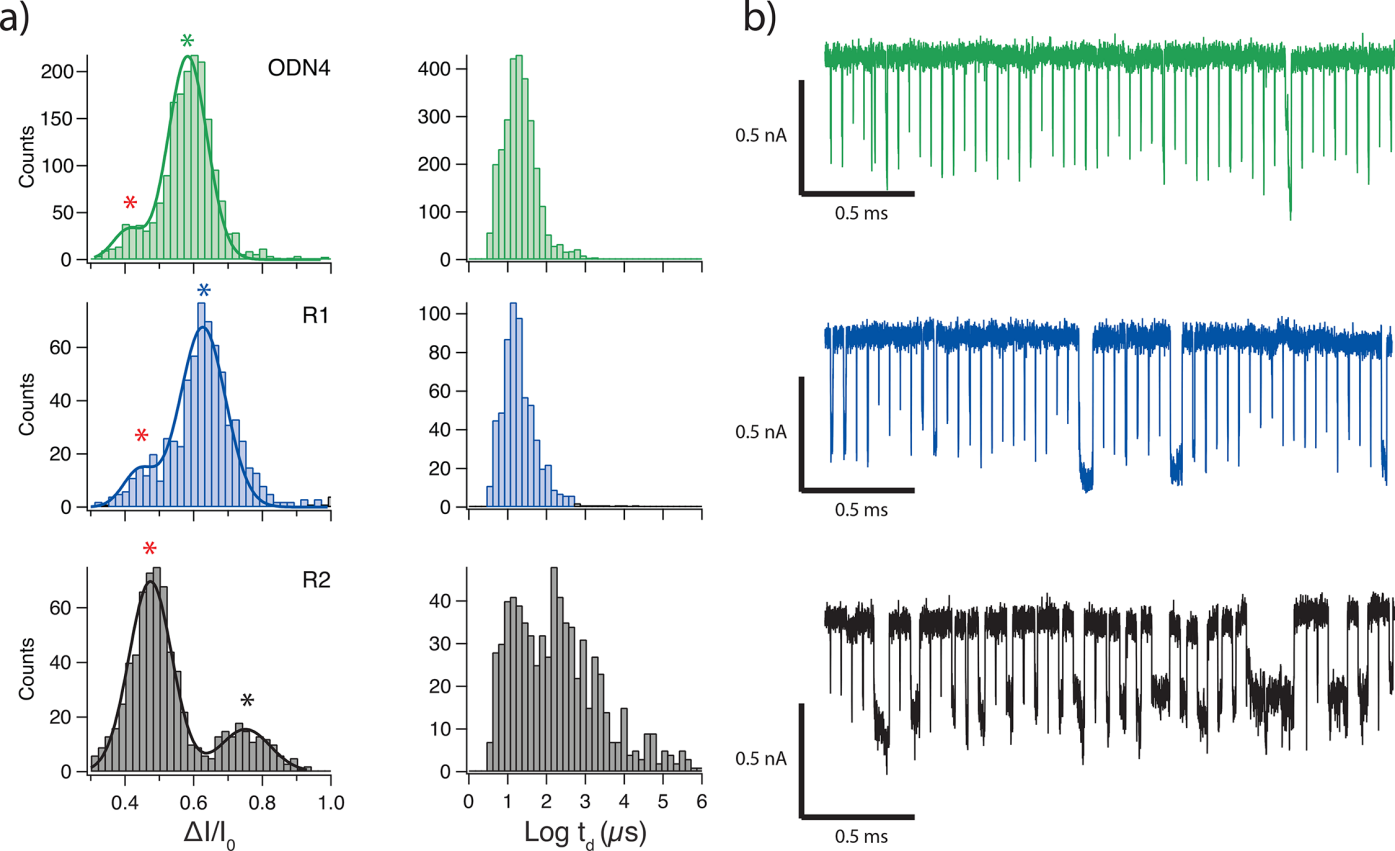
Translocation events for an 80-mer fragment after R1 and R2 reactions. **(a)** Histograms are shown for the fractional current blockade as well as the dwell times for 80-mer ssDNA with varying degrees of osmylation passing through a SiN nanopore with an applied bias voltage of 300mV. T+C osmylated molecules show markedly greater dwell times as compared to unreacted and T-osmylated molecules. The red stars indicate all of the lower blockade peaks of the double Gaussian fits, which correspond to collision events; green, blue, and black stars indicate the locations of the higher blockade translocation peaks for each distribution. **(b)** Concatenated events are shown for each molecule. Data is shown after low-pass filtering at 100 kHz.

We used a SiN nanopore with diameter = 1.6 nm and effective thickness = 3 nm (nanopores were sized as previously described [16]) to translocate unreacted 80-mer ssDNA, molecules that had been T labeled (R1), and molecules with T and C labeled bases (R2). Roughly 1,000 translocation events were collected for each at an applied voltage of 300 mV. **Fig 2A** shows *ΔI/I*_*o*_ and *t*_*d*_ histograms for the 80-mer fragment with varying degrees of osmylation, and **Fig 2B** shows sample concatenated events for each. Each *ΔI/I*_*o*_ histogram displays a double Gaussian distribution, where the location of the lower blockade population seems to be unchanging; accordingly we fit each to a double Gaussian curve. This lower blockade peak (the left most peak for each histogram in **Fig 2A** has an average value of *ΔI/I*_*o*_ = 0.44 ± 0.04. This group likely corresponds to molecules colliding with the pore. The frequency of this peak logically increases as the size of the molecule increases, arising from an increased steric hindrance to the passage of the molecule. The larger blockade peak (the right most peak for each histogram in **Fig 2A**) likely corresponds to the translocation events for each molecule, as it logically becomes less frequent with a wider, more osmylated molecule, but also blocks more as the osmylation level increases. The location of these peaks suggests a trend of increasing *ΔI/I*_*o*_ as a function of higher level of osmylation, while the dwell time histogram does not appear to shift significantly upon T-osmylation. The T+C osmylated molecule is quite different as the distribution seems to develop a second population that stretches several orders of magnitude. These longer dwell time events correspond to the lower *ΔI/I*_*o*_ collision events (see S1 **Fig**) indicating that a translocating ssDNA strand’s level of osmylation can be qualitatively inferred from its *ΔI/I*_*o*_ level. Further, this serves as a proof of principle for osbipy modification as a viable contrast agent for nanopore based DNA base identification.

Next, we tested this system’s ability to differentiate varying levels of osmylated base content. To do this we used three 20-mer oligos with varying degrees of T and C content, and osmylated each at two levels (i.e., T only or T+C). A list of all ssDNA samples used in this paper is presented in **Table 1**.

**Table 1:** List of oligo sequences, osmylation levels, and current blockades.

Oligo ID	Sequence (length)	R (312nm/272nm)	Modification	Osbipy #	ΔI/I_ο_
ODN1	CGC GAA GTG GAG CAG CCT GG (20)	0.03	None	0	NA/ 0.583
ODN1 (R1)	CGC GAA G**T**G GAG CAG CC**T** GG (20)	0.24	T-osmylation	2	NA/ 0.582
ODN2 (R1)	AGG **T**GA GA**T** GAC AGG AGA **T**C (20)	0.32	T-osmylation	3	NA/ 0.624
ODN3 (R1)	AGA G**TT T**GA **T**CC **T**GG C**T**C AG (20)	0.64	T-osmylation	6	0.60/0.667
ODN3 (R2)	AGA G**TT T**GA **TCC T**GG **CTC** AG (20)	1.05	(T+C)-osmylation	10	0.58/0.69
ODN4	CTC AGA GTT CCA AGG TGA GTG AGT GAG TGG TCC TTC CTT CCT TCC TTC CGG TGA GTG AGT GAG TGG ACG GTA AGC CAT TT (80)	0.03	None	0	0.42/0.57
ODN4 (R1)	C**T**C AGA G**TT** CCA AGG **T**GA G**T**G AG**T** GAG **T**GG **T**CC **TT**C C**TT** CC**T T**CC **TT**C CGG **T**GA G**T**G AG**T** GAG **T**GG ACG G**T**A AGC CA**T TT** (80)	0.60	T-osmylation	24	0.43/0.63
ODN4 (R2)	**CTC** AGA G**TT CC**A AGG **T**GA G**T**G AG**T** GAG **T**GG **TCC TTC CTT CCT TCC TTC CGG T**GA G**T**G AG**T** GAG **T**GG A**C**G G**T**A AG**C C**A**T TT** (80)	1.18	(T+C)-osmylation	41	0.46/0.74

Osmylated bases represented in bold face. Percent purity determined by capillary electrophoresis (CE) in the range of 89–93%; purity of osmylated oligo comparable to that of the starting material. R (312 nm/272 nm): the ratio of the peak area at the two different wavelengths (see Materials and Methods) [6]. R1 or R2 describes material obtained following Protocol A or B, respectively. Most probable value for *ΔI/Io* is indicated, a single value is given for those where fractional current blockade histograms were fit to Gaussian curves, two values are given for those obtained by fitting to double Gaussian curves.

**Fig 3:**
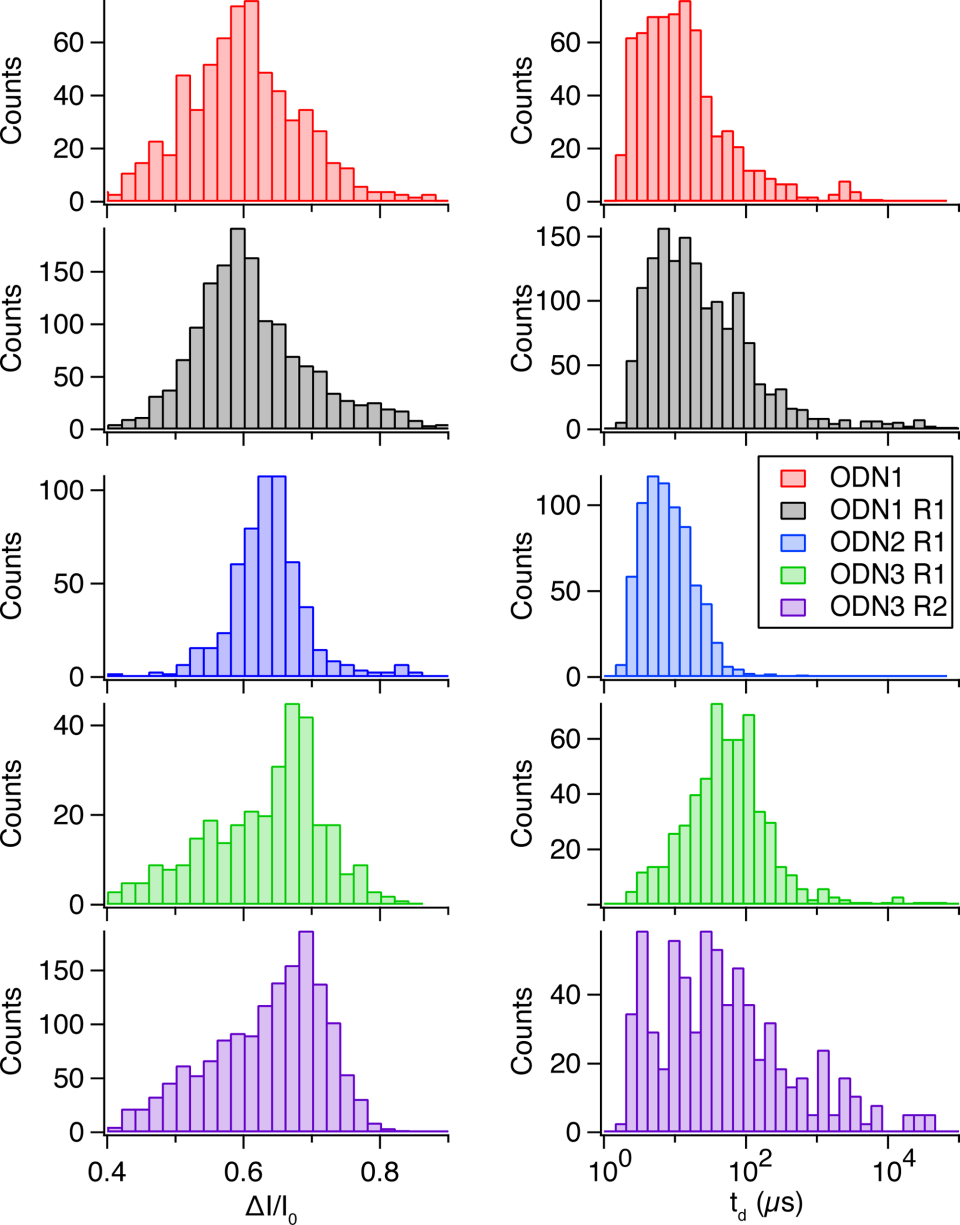
Translocation statistics for 20-mer ssDNA with various osmylation levels. Histograms of the fractional current blockade (left) and the log of the dwell time (right) for several 20-mer single-stranded DNA samples (see text). The fractional current blockades increase with the degree of T-content upon osmylation, while similarly; dwell times increase with osmylation levels.

For this study we performed nanopore translocation experiments with each of these samples using a 1.6 nm diameter pore with an effective thickness of 10 nm. A bias voltage of 200 mV was applied across the pore and 600–1,200 translocation events were collected for each of the samples. Histograms of the *t*_*d*_ and *ΔI/I*_*o*_ values for of each event can be seen in **Fig 3**. Among the 20-mer samples, the three least osmylated samples display *ΔI/I*_*o*_ distributions that are well approximated by single Gaussian distributions. ODN1 and ODN R1 *ΔI/I*_*o*_ distributions do display a tail towards larger distributions, this is likely explained by various degrees of DNA folding and self-interaction during translocation, though the urea present in the buffer minimizes the effects of DNA self interaction. As the degree of osmylation increases the tail in the distributions seems to disappear, possibly due to the bulky osbipy groups sterically hindering the flexibility of the DNA and increasing its persistence length. As the degree of osmylation further increases a smaller second population forms at lower blockade levels. Accordingly, we report two peak values for ODN3 R1 and ODN3 R2 populations fit to double Gaussian distributions (indicated in **Table 1**). This less frequent population may arise from an increased probability of collision events for more osmylated molecules, as seen with the R2 80-mer molecule (see **Fig 2**). The peaks of the Gaussian curves for each distribution are listed in **Table 1**. Notably, the corresponding *ΔI/I*_*o*_ of the largest peak increases with increased osmylation.

**Fig 4:**
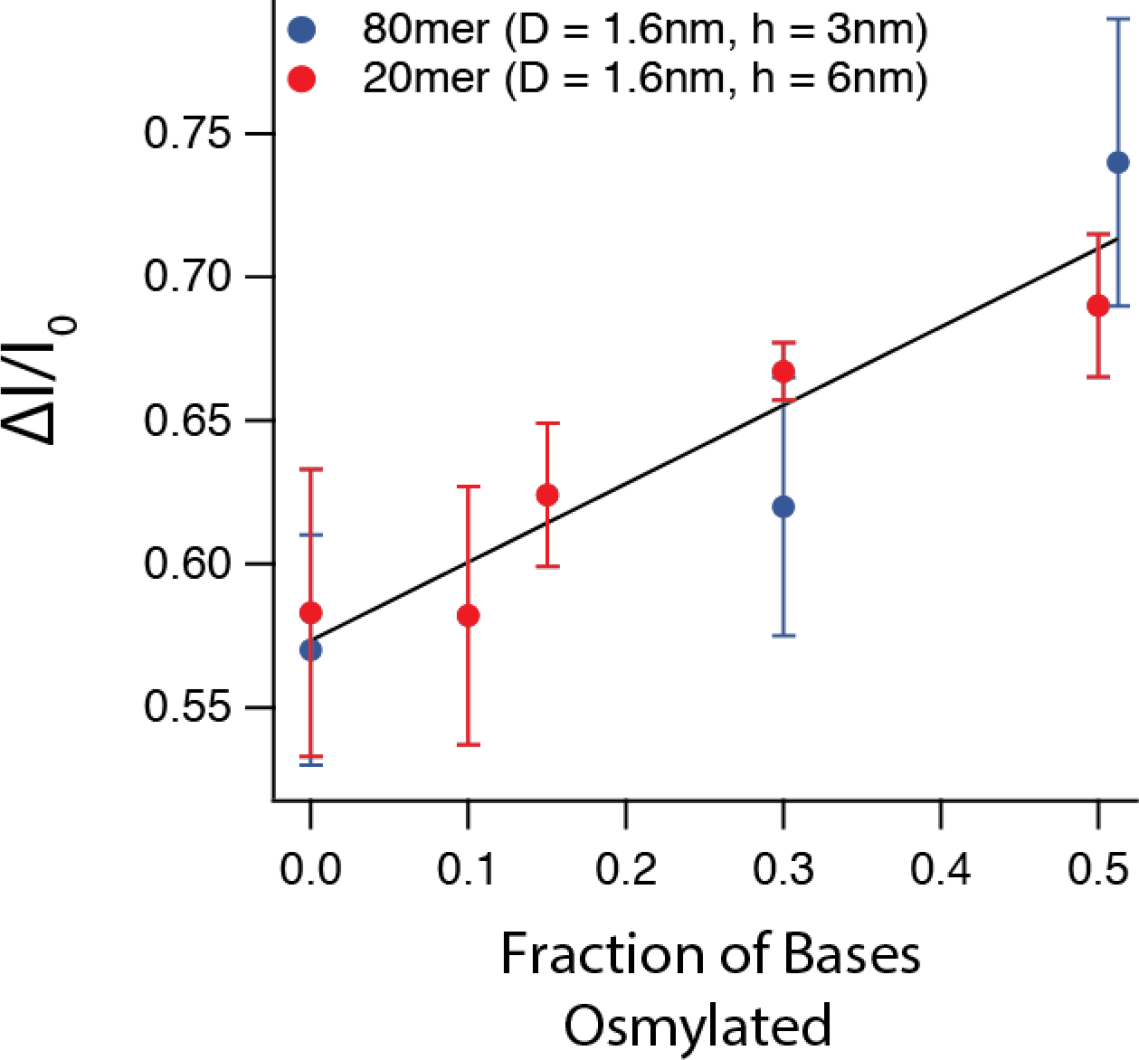
Ion current blockade values scale with osmylation. Fractional current blockade vs. fraction of osmylated bases. Two data sets are shown (20-mer in red and 80-mer in blue) and a linear trend line is shown to guide the eye, but does not imply any physical model.

The extended length of a 20-mer DNA strand is ~6.4 nm, and therefore the signal from the 6-nm-thick pore is an average of the entire strand. The ability to detect these small modifications is modulated by the thickness of the substrate, and could be greatly improved with the use of ultrathin substrates [16]. Despite this, the amount of current blocked by a translocating DNA molecule clearly increases as the number of osmylated bases increases. **Fig 4** displays the most probable *ΔI/I*_*o*_ values from all of the translocation peaks presented thus far. Values for *ΔI/I*_*o*_ were obtained from the peaks of the Gaussian fits; error bars correspond to standard deviation of the fit. These results suggest that if one were able to regulate the motion of DNA through the pore, they would be able to discriminate between osmylated and unosmylated bases with efficiency much higher than simply detecting the differences between native bases.

Osmylation of DNA bases has also been used as a strategy for studying dsDNA [12]. Though these studies have mainly focused on the use of the osbipy complex to probe large structural distortions in DNA structure. As addition of the Osmium tag at the C5-C6 double bond of the pyrimidines does not interfere with the base-pairing side of the ring, we developed (see Materials and Methods) an osmylation protocol specifically for dsDNA that presumably preserves the double-stranded structure. For this purpose we prepared dsDNA fragments with a length of 202 bp (see **Materials and Methods** for sequence) using standard PCR protocols. Next, we subjected the fragments to a modified protocol to ensure osmylation of ~50% of thymidine bases (as confirmed by capillary electrophoresis). Of the 202 bp, ~55% were AT pairs, a 50% osmylation of T bases would result in 27.5% strand osmylation, or ~55 osmylated base pairs.

**Fig 5:**
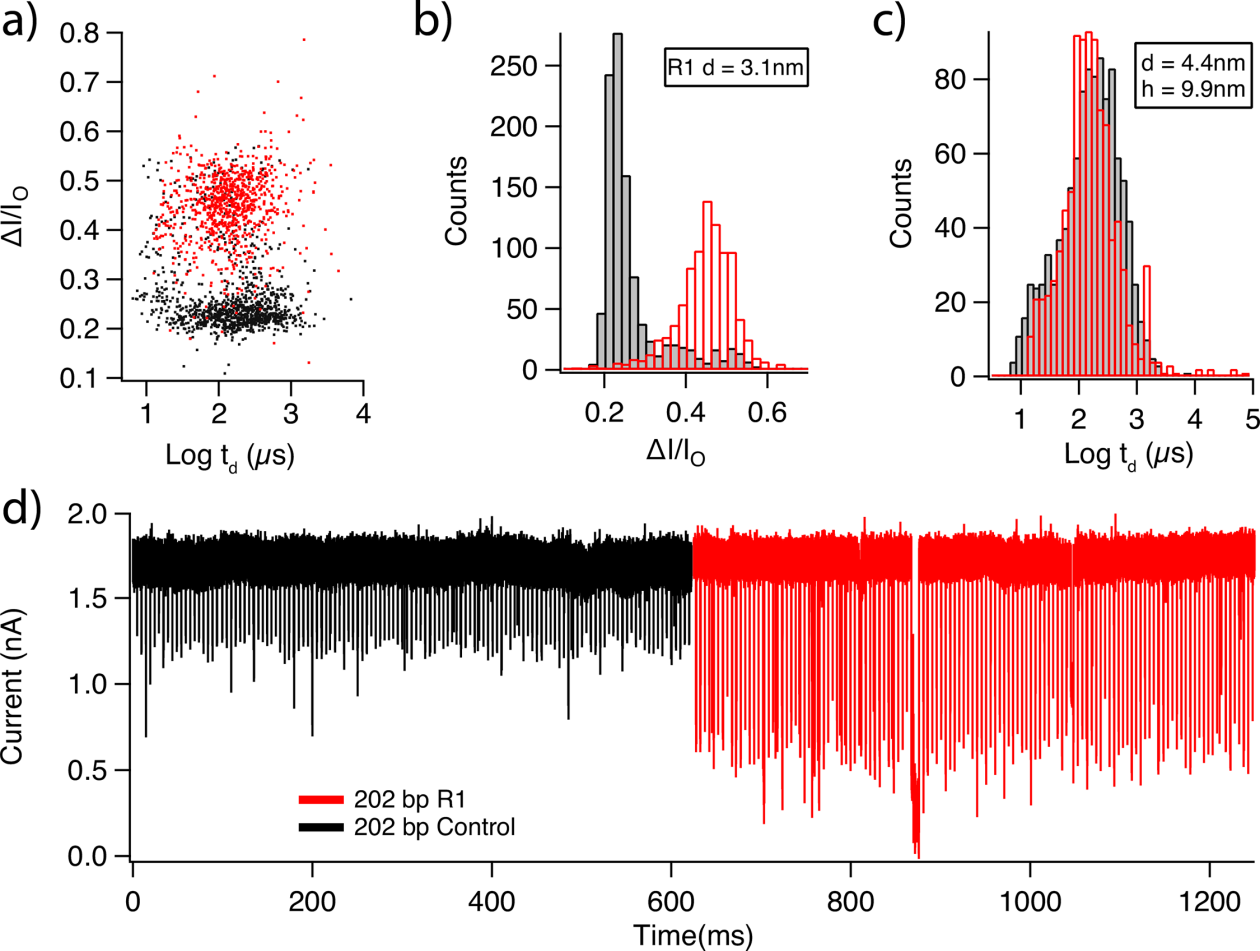
Detection of osmylated dsDNA. **(a)** A scatter plot is shown for the dwell time and fractional current blockade values for the translocation of unlabeled 202 bp dsDNA (black) and osmylated 202 bp dsDNA (red) molecules through a SiN nanopore (diameter = 4.4 nm, effective thickness 10 nm, applied bias = 300 mV). **(b)** Histograms for the fractional blockade values from each molecule are shown. R1 molecule displays a peak ΔI/I_o_ = 0.44. This corresponds to a molecule with a diameter of 3.1 nm when fit to our conductance models. **(c)** Dwell time histograms are shown for both molecules. **(d)** Concatenated events are shown with 20,000 baseline data points separating each event. Data is shown after low pass filtering at 200 kHz.

We conducted dsDNA nanopore experiments using nanopores measuring 4.4 nm in diameter and 10 nm in effective thickness. For these experiments the urea was removed form the buffered solution (0.40 M KCl, 1 mM EDTA, and 10 mM Tris, at pH 8.0). We collected >1,000 translocation events for both the osmylated and unosmylated molecules separately. Statistics for these events are presented in **Fig 5**. The osmylated molecule can be clearly seen to block substantially more than the unosmylated molecule. When fit to Gaussian distributions the bare 202 bp molecule displays a peak at *ΔI/I*_*o*_ = 0.22, while the osmylated molecule fits to a peak value of *ΔI/I*_*o*_ = 0.44. Using the simple geometric nanopore conductance models described previously [15], and assuming the geometry previously obtained by sizing the pore with bare dsDNA, we obtain an average effective diameter of ~3.1 nm for the osmylated molecule. Notably, the dwell time distributions display no discernable shift after osmylation, indicating that duplex osmylation does not appreciably hinder its transport. The combination of these results suggests that the use of osmylation as a contrast agent may prove useful in future genome mapping applications.

## Discussion

We have shown here that SiN nanopores with diameters of ~1.6 nm can be used to detect the presence and relative concentration of osmylated bases along individual ssDNA strands. The relative concentration of osmylated bases increases the amount of current a DNA strand blocks as it translocates the pore, while also increasing the time required for the strand to translocate in cases of highly osmylated molecules. The current blockade enhancement, apparent for both T osmylated and T+C osmylated samples, serves as a basic proof of principle for contrast enhancement from specific bases, a step towards error reduction during nanopore-based DNA sequencing. And in its coarsest version, this facile technique may be used to quickly assess the T or C richness of a sequence.

We have also shown that partial osbipy modification of thymine bases is a viable technique for increasing the contrast of nanopore translocation signals from dsDNA. Osmylation increases the effective dsDNA diameter from ~2.2 nm to ~3.1 nm, while showing no signs of inhibiting the translocation process for a 4.4 nm diameter nanopore, and thus may be a promising tool for genome mapping. This genome mapping technology may in principle be combined with the aforementioned nanopore sequencing techniques into a single device capable of complete de novo genome assembly.

Moving forward, we propose that the use of thinner membranes such as hafnium oxide [17], graphene [18–20], or molybdenum disulfide [21–23] may provide the ability to detect individual osmylation sites as single inter-event current spikes. In addition it may be possible to couple the system described here with a DNA ratcheting enzyme to ratchet osmylated oligos through nanopores as and improve upon existing DNA sequencing technologies [3, 4]. For systems that do no involve the use of enzymes salt concentrations much higher than 0.4 M may be used, increasing signal-to-noise ratio and potentially allowing for read out of individual osmylated sites.

## Materials and Methods

### Preparation of Osmylated Oligos, and Capillary Electrophoresis (CE) Analysis

HPCE grade solution of 50 mM sodium tetraborate pH 9.3 was purchased from Agilent Technologies. A 4% aqueous osmium tetroxide solution was purchased from Electron Microscopy Sciences. 2,2’-bipyridine 99+% (bipyridine) was purchased from Acros Organics. Deoxyoligonucleotides (Oligos) were purchased from Integrated DNA Technologies (IDT), diluted with DNase/RNase-free water (from MP Biomedical) to 1 μg/mL and stored at -20°C. The purity of these oligos was tested using CE in 50mM sodium tetraborate at pH 9.3 (see below). Oligos used in this study, their sequences, and the osmylation products of these oligos are listed in Table 1.

Aqueous reaction mixtures were prepared with DNase/RNase-free water. A stock solution of Osbipy at 15.75 mM (OsO_4_: bipyridine = 1:1) was prepared by mixing bipyridine in OsO_4_ solution. This stock solution was dispensed in small glass vials, sealed with parafilm, and kept at -20°C until use; no detectable change in reactivity was observed with solutions that were stored sealed at 4°C for two weeks. Experiments were initiated by mixing the Osbipy stock solution and the Oligo stock solution directly in a CE glass vial filled with the appropriate amount of distilled water. Osbipy was at a large excess of 20-fold or more compared to the Oligo. No buffer was added and reaction mixtures were incubated at room temperature. Protocol A was used for osmylating all T’s, and Protocol B for osmylating all T’s and C’s in an Oligo. Protocol A is conducted by 60 minutes incubation with 3.15 mM Osbipy and Protocol B with 12 hours incubation with 12.6mM Osbipy. Purification to remove extra label was conduced immediately after incubation. Spin columns TC-100 TC-FC from TrimGen were used to remove excess Osbipy according to the manufacturer’s instructions. Practically 100% recovery of labeled Oligo and removal of up to 15 mM Osbipy down to detectability levels after two rounds was achieved. As elaborated elsewhere Protocol A results in 90% T-osmylation and 6.5% C-osmylation and Protocol B results practically to 100% both T- and C-osmylation for each oligo tested ([6, 7], and pending patent # 62/083,256).

Analyses of the osmylation products of the Oligos were carried out using an Agilent 1600 CE instrument equipped with Diode Array Detector (DAD) and Chemstation software, Rev.B.04.02SP1, for data acquisition and processing. Untreated fused silica capillaries (50mm x 40cm) with extended light path were purchased from Agilent Technologies.

Analysis of reaction mixtures was conducted using Capillary Zone Electrophoresis method in 50mM sodium tetraborate pH 9.3 with 20 kV. The capillary’s temperature was matched to the autosampler’s temperature and ranged from 25 to 27°C. A typical method was 18 min long and included a 4 min automatic buffer conditioning ahead of sample analysis. Osbipy migrates at about 3 min and oligos/DNA at about 10 min. Osbipy-labeled products migrate between the Osbipy peak and the corresponding Oligo peak. Resolution between starting material and osmylated product, is among other parameters, a function of the length and the number of reacting bases. With the tested Oligos, a shift of the peak to earlier migration is detected as a function of reaction progress. Peaks on CE were detected and identified using the DAD in the UV-vis region 200 nm to 450 nm and the electropherograms were recorded at several wavelengths including 272 nm and 312 nm. In contrast to DNA that exhibits no detectable absorbance at 312 nm, osmylated DNA absorbs at 312 nm. For identification purposes we abbreviate R1 (312/272) the ratio of the absorbance at the two wavelengths for the product of the reaction following Protocol A, and R2 (312/272) the corresponding ratio following Protocol B.

The 202 bp dsDNA sample was prepared by polymerase chain reaction (PCR) using HeLa genomic DNA (purchased from New England Biolabs) as a template. The sequence for the sample used is as follows: **TTGGTAGCAAAAACGAACTGGGT**TCAATCTTGGCTCTGCCACCCATTCCTGGACAAAATACTTAACTCTCTCCATCTTCCAAATGTGACCTACACCTTACAGGACTGCCAGGAGAATTAAGGAAGCACATACACAGTGCCTGACACACAAGTGAGTAAACACCTTAAACTATGTTTAGCCA**CAGAACCAGAGTGGTTGGAGG** (primer regions are shown in bold). The PCR products were purified using Biobasic EZ-10 spin column purification kit. The purity and quantity of the recovered DNA was then evaluated spectrophotometrically at 260 and 280 nm. The 202 bp dsDNA was osmylated with a proprietary protocol that presumably leaves the ds structure intact, while osmylating only about 50% of Ts in both strands (patent pending, # 62/212,072). Evidence for undistorted ds structure is obtained by CE analysis, as ssDNA migrates ahead of dsDNA; the same order is also valid for their osmylated conjugates.

### Nanopore data acquisition and analysis

Data was acquired at 4.16 MHz using a Chimera Instruments VC100 and low-pass filtered at 250 kHz to provide a suitable signal-to-noise ratio for event detection. Current blockage and dwell time analysis of the traces was performed using Pythion, custom software written by our group (https://github.com/rhenley/Pyth-Ion/).

## Supporting Information

S1 Fig Contour plots displaying fractional current blockade and dwell time values for 80mer ssDNA molecules with varying levels of osmylation. Control molecule contains no osmylated bases. R1 molecules have been T-osmylated, with 24/80 total bases being modified. R2 molecules are T+C modified, with 41/80 total bases being modified.
(EPS)
